# Peter Houghton (1947 – 2024) – in memoriam*

**DOI:** 10.1080/13880209.2024.2365210

**Published:** 2024-07-01

**Authors:** Michael Heinrich

**Affiliations:** Research Group ‘Pharmacognosy and Phytotherapy’, UCL School of Pharmacy, University in London, London, UK


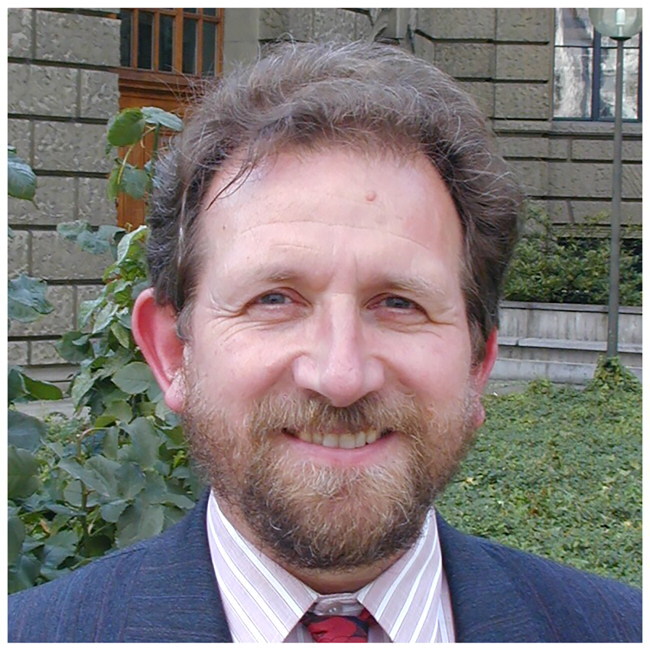


On 25 April 2024, Prof. Peter Houghton, Emeritus Professor of Pharmacognosy at Kings College London, passed away at the age of 77 following a stroke. He had recovered well from melanoma which had also spread to the brain, but then further health challenges developed. Here we remember him most importantly as a long-term associate editor of the journal *Pharmaceutical Biology*.

As a pharmacist, he was passionate about medicines, especially everything related to plants. Importantly, he was one of the most caring and supportive academic teachers one can imagine. In 2008, he took early retirement and became a vicar of the Church of England, a role he fulfilled with as much love and dedication as he did previously as an academic (Heinrich 2009). In the last years his wife Joan and he lived in Hereford in the East of England, where he continued to work as a vicar, until he retired from this position, too.

Peter came to London to study pharmacy at the Chelsea College (of Pharmacy), which was later merged into Kings College London, and in 1968 graduated with a Bachelor of Pharmacy (BPharm). After his pre-registration training as a pharmacist in Cambridge, Peter returned to Chelsea College and developed a career working at the college and later King’s College (and on planes to all remoter regions of the world). In 1973 he was awarded his PhD and, since 1972, lectured and undertook research in pharmacognosy and specifically ethnopharmacology. He was made Professor in Pharmacognosy in 1999 and in December 1994 was designated a Fellow of the Royal Pharmaceutical Society of Great Britain (RPSGB) and became a fellow of the Royal Society of Chemistry. He was the president and long-term treasurer of the International Society for Ethnopharmacology. For many years he served as in editorial positions of the journals *Pharmaceutical Biology* and the *Journal of Ethnopharmacology* and, from 1999 until 2008, he was a board member of the Society for Medicinal Plant and Natural Product Research (GA). In addition, he served as an editorial board member of many other journals, including the *Journal of Pharmacy and Pharmacology, Planta Medica* and *Phytochemical Analysis.* Further, for many, he is remembered for his admiration and curiosity about elephants.

Peter published over 250 research papers and reviews on many topics connected with the chemistry and biological activity of plants and their metabolites. This includes substances from plants of potential use in treating CNS degenerative disease, cancer and for wound healing [e.g., Houghton (1999), Howes & Houghton (2003) and Mensah et al. (2006)]. One of his core interests has always been the adaptation and implementation of biological and biochemical-pharmacological *in vitro* test methods for use with plant extracts (*cf*. Houghton et al. 2007). Especially during the early years of his career, he focused on the genus *Buddleja* and made many important contributions to the chemistry of the genus and the *in vitro* pharmacological effects of the extracts (Houghton et al. 2003). He also wrote/co-edited several books (e.g., Prendergast et al. 1998). For many years he organised a pharmacognosy workshop at the British Pharmaceutical Conference, later of the symposia of the Academy of Pharmaceutical Sciences of Great Britain (APSGB), and was involved in the organisation of many other symposia.

A publication record says a lot and he supervised numerous PhD students who went to positions all over the world. At this moment we remember him as a wonderful person and colleague. Many of my former PhD students remember him as a warm, meticulous and very supportive supervisor and similarly when he acted as an (external) examiner during their PhD viva. He was an external examiner for many universities well beyond the UK.

**In Amala Soumyanath’s (nee Raman) words (Oregon Health & Science University, USA):** “Needless to say, Peter was a great mentor to me during my early days as a faculty member [at Kings College]. During the period 1990 to 2002, Peter and I worked to modernize the pharmacognosy curriculum to meet these pressures and adapt to the changing role of the pharmacist (including advising on botanical OTC products) and pharmacy education (BPharm to MPharm).”

Here some further personal snippets and recollections:

**Melanie-Jayne Howes (Royal Botanic Gardens, Kew, London, UK):** “I was taught by Peter as a Pharmacy Undergraduate at King’s College London, where his knowledge and enthusiasm for phytochemistry and pharmacognosy inspired me to follow a career in these areas of research. Peter later became my PhD supervisor in the Pharmacy Department at King’s College London. This research led to the first plant diterpenoids to be isolated as potent inhibitors of the enzyme acetylcholinesterase, and the GlaxoSmithKline Prize for Best PhD Thesis in the Pharmacy Department. This was the start of continued collaboration with Peter on natural products relevant to central nervous system disorders, resulting in many joint publications. Peter had great scientific integrity and was always supportive. It was an honour to have been taught and supervised by him as a student, and to have been mentored by him throughout my career.”

**Cate Whittlesea (UCL School of Pharmacy, London, UK): “**I have such fond memories of working with Peter at Kings College London and as a PhD student demonstrating for his undergraduate practical classes. My thoughts and prayers are with his family at this sad time.”

**Namrita Lall (University of Pretoria, South Africa):** “I will always remember him for his kindness. His advice and insights have been instrumental for the career growth for many of us and I am deeply grateful for the time and effort he invested in mentoring myself and also my students.”

**Pulok Mukherjee (Institute of Bioresources and Sustainable Development – IBSD, Imphal, India):** “He was my mentor, guide and a very wonderful friend. I have known him for over 25 years- we first met at Leiden, and later on I worked with him at Kings College Waterloo Campus as a Commonwealth Fellow. His company will be deeply missed, so inspiring always and helped a lot to many academics in India. We have very large groups of his students who are in different positions.”

**Doug Kinghorn (Ohio State Univ., USA):** “During his career, he made substantial phytochemical contributions to ethnopharmacology, and was an accomplished scientific writer. I had the pleasure of his company when returning to London from the joint GA-SIF Congress held in Florence, Italy, in 2005, and I found him to be a delightful traveling companion.”

**Judith Rollinger (University of Vienna, Austria):** “I remember well – some 17 years ago – he invited me for a lecture in his department at Kings College London. It was such a kind and warm-hearty meeting with an excellent scientist and lovely person.”

**Clara Lau (University of Hong Kong, HK): “**I will never forget the summer project (at KCL) and the final year research project under his supervision, resulting in a poster presentation in the BPC conference in 1993. I still remembered that day he drove me to Reading to attend the first international conference in my life; what an unforgettable experience to a BPharm graduate. In fact, he was the one who brought me into the fascinating pharmacognosy world and has great influence and support on my entire academic career. I am blessed to have had him as my teacher, and I will always remember his guidance, kindness and laughter.”

So many more personal recollections could be included, and I personally would like to add, that without this great generous and wonderful colleague, I presumably would not have moved to London with my family. I will never forget, in late 1998, he told me that a position was opening at ‘The Square’ (School of Pharmacy, Univ. London) and a few weeks later he sent a fax with the advert, with a short note added: “Don’t be surprised if we meet at the interview, since it has been advertised at such a senior level, that, I, too, will apply.” We both applied, and after I moved to London, we were close collaborators and friends until his untimely death.

What a great heart, brain, and spirit. Not only elephants are recognized for their *wisdom, strength, and great courage.* Yes, we miss him and there are so many people who are grateful for his support, guidance, academic leadership, and ultimately love for people and plants.

**Figure:**
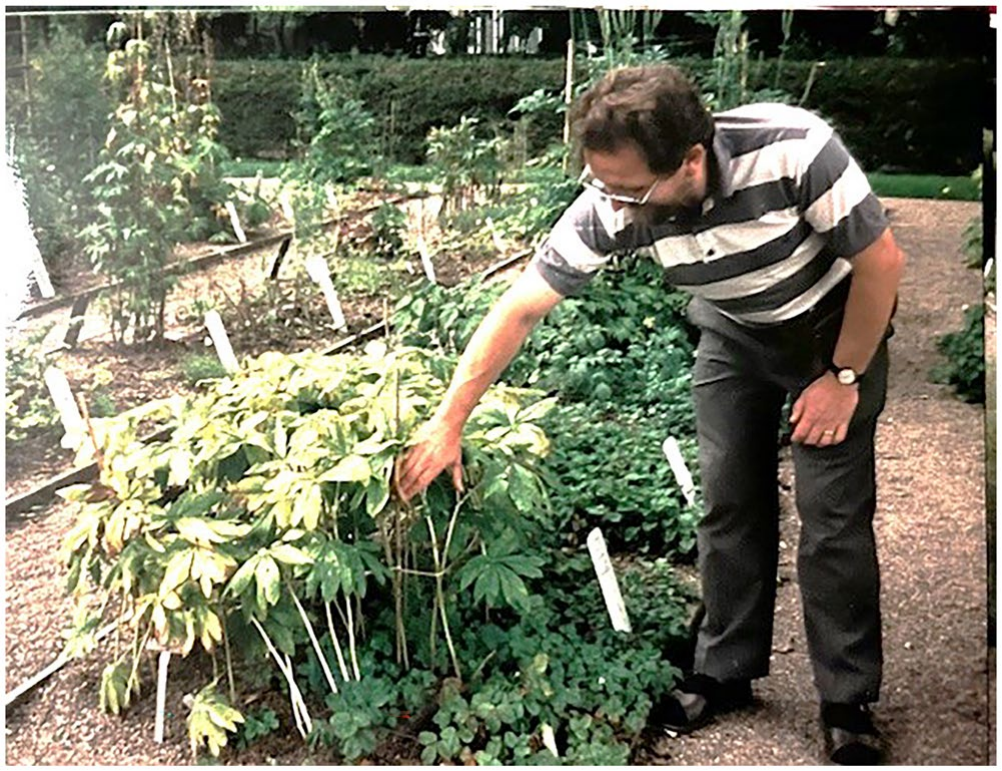
Photograph of Prof. Peter Houghton (*ca*. 2000) in Zurich, Switzerland (above) and Photograph of Peter Houghton at the Chelsea Physic Garden.
